# Key Molecular Mechanisms of Chaiqinchengqi Decoction in Alleviating the Pulmonary Albumin Leakage Caused by Endotoxemia in Severe Acute Pancreatitis Rats

**DOI:** 10.1155/2016/3265368

**Published:** 2016-06-19

**Authors:** Wei Wu, Ruijie Luo, Ziqi Lin, Qing Xia, Ping Xue

**Affiliations:** Department of Integrated Traditional Chinese and Western Medicine, West China Hospital of Sichuan University, Chengdu 610041, China

## Abstract

To reveal the key molecular mechanisms of Chaiqinchengqi decoction (CQCQD) in alleviating the pulmonary albumin leakage caused by endotoxemia in severe acute pancreatitis (SAP) rats. Rats models of SAP endotoxemia-induced acute lung injury were established, the studies in vivo provided the important evidences that the therapy of CQCQD significantly ameliorated the increases in plasma levels of lipopolysaccharide (LPS), sCd14, and Lbp, the elevation of serum amylase level, the enhancements of systemic and pulmonary albumin leakage, and the depravation of airways indicators, thus improving respiratory dysfunction and also pancreatic and pulmonary histopathological changes. According to the analyses of rats pulmonary tissue microarray and protein-protein interaction network, c-Fos, c-Src, and p85*α* were predicted as the target proteins for CQCQD in alleviating pulmonary albumin leakage. To confirm these predictions, human umbilical vein endothelial cells were employed in in vitro studies, which provide the evidences that (1) LPS-induced paracellular leakage and proinflammatory cytokines release were suppressed by pretreatment with inhibitors of c-Src (PP1) or PI3K (LY294002) or by transfection with siRNAs of c-Fos; (2) fortunately, CQCQD imitated the actions of these selective inhibitions agents to inhibit LPS-induced high expressions of p-Src, p-p85*α*, and c-Fos, therefore attenuating paracellular leakage and proinflammatory cytokines release.

## 1. Introduction

Over the natural course of severe acute pancreatitis (SAP), intestinal permeability has been found to increase within the first 72 hours, which led to massive endotoxin, also known as the lipopolysaccharide (LPS) aggregates, to be uptaken into blood circulation which initiates the occurrence of endotoxemia [1]. Previous study has described that endotoxemia would initiate the consistent proinflammatory changes in genic and proteinic expression profile of the immune cells, which would trigger inflammatory cascade reaction and then cause the serious dysfunction of capillary endothelium [2]. Because pulmonary capillary networks are extremely enriched, in the early stage, endotoxemia would initiate the severe damage to pulmonary capillary endothelial barrier and then cause acute lung injury (ALI) [3]. The analyses of SAP-induced extrapancreatic organs injury indicated that ALI not only occurs at the earliest stage but also initiates the most serious injury among these extrapancreatic organs [3]. The SAP endotoxemia-induced ALI would directly lead to a significant decrease in ventilation/perfusion ratio, which eventually result in respiratory failure [4]. And the incidence of ALI in SAP patients is 20%, and the mortality rate is as high as 15% to 25% [4]. Therefore, alleviating SAP endotoxemia-induced ALI could decrease the mortality rate in SAP. The physiopathologic mechanisms of the damage to pulmonary capillary endothelial barrier mainly include the disassembly of tight junction (TJ) and adherens junction (AJ) and reorganization of cytoskeleton, which would enhance pulmonary albumin leak and then result in the extra-vascular leak of the increased albumin leak-induced edema fluid into pulmonary interstitial and alveolar compartments and eventually initiate respiratory failure [5, 6]. Hence, pulmonary albumin leakage is the key pathophysiological mechanism of ALI. Additionally, albumin with high molecular weight would leak across capillary endothelial barrier mainly dependent on the paracellular leakage [7]. Therefore, the enhancement to systemic and pulmonary albumin leak, depravation of airways indicators, respiratory dysfunction, and also the adverse change in lung tissue pathology could be considered as the important features of pulmonary capillary endothelial barrier injury [8–10]. Moreover, the reversal of the enhanced pulmonary albumin leak should be considered as the therapeutic goal for ALI caused by endotoxemia in SAP.

CQCQD decoction, a famous Chinese medicinal formula, has been used to treat SAP for decades in West China Hospital, Sichuan University [11]. According to the regulations on drug development and approval of SFDA, before the Chinese medicinal formula is widely used in clinical practice, there are four main principles required to be met by the Chinese medicinal formula: (1) action target area; (2) highly efficient concentration; (3) efficacy in animal model; (4) multicentral random clinical trial. The growing studies evidences of CQCQD indicated that the first three principles mentioned above have been met, which will be shown as follows: (i) action target area: (1) pancreatic tissues, (2) gastrointestinal tract, (3) lung, (4) immune system, (5) pancreatic acinar cells, (6) alveolar macrophages, and (7) peritoneal macrophages [12–25], which suggested that the lungs are the important action target area for CQCQD. (ii) Highly efficient concentration in clinical practice is as follows: CQCQD consists of* Bupleuri Radix* (Chai Hu) 15 g,* Scutellariae Radix* (Huang Qin) 15 g,* Magnoliae Officinalis Cortex* (Hou Pu) 15 g,* Aurantii Fructus Immaturus* (Zhi Shi) 15 g,* Artemisiae Scopariae Herba* (Yin Chen) 15 g,* Gardeniae Fructus* (Zhi Zi) 20 g,* Rhei Radix et Rhizoma* (Da Huang) 20 g, and* Natrii Sulfas* (Mang Xiao) 20 g [12]; according to the standardized preparation procedure of CQCQD formula, these eight herbs will be decocted to form this decoction [13]; since this decoction has been used to treat SAP for decades and the effective pharmacotherapy to the patients has been certified with ALI caused by endotoxemia in SAP [14], if this decoction is prepared strictly according to CQCQD formula and the standardized preparation procedure, the final concentration of the prepared decoction can represent the highly efficient concentrations of CQCQD in clinical application [15]. (iii) Efficacy in SAP animal model is as follows: (1) effectively ameliorating pancreatic histopathological changes and systemic inflammatory response [16–23]; (2) effectively improving gastrointestinal motility dysfunction [23]; (3) effectively ameliorating alveolar inflammatory response [24]; (4) effectively alleviating ALI [25], which suggested that the therapy of CQCQD could effectively alleviate the ALI caused by endotoxemia in SAP animal model. Meanwhile, according to the 4th principle, multicentral random clinical trials are being conducted in a number of hospitals in China, including West China Hospital of Sichuan University, Chengdu No. 1 Hospital, and Sichuan Hospital of TCM. Although the multicentral random clinical trials have not been completed, a large number of clinical studies have provided the key evidences that (1) the therapy of CQCQD significantly decreased serum levels of interleukin-6 (IL-6) and IL-1*β* receptor antagonist (IL-1ra) in SAP patients and also the hospitalization time of SAP patients [12]; (2) the therapy of CQCQD could decrease the serum levels of matrix metalloproteinase-9 (MMP-9), C-reactive protein (CRP), and amyloid A (SAA) to relieve the durations of acute respiratory distress syndrome (ARDS) in patients with SAP [13, 14], and the early application of CQCQD is more effective than late application to shorten the duration of ARDS [15], which suggested that the therapy of CQCQD could probably effectively alleviate ALI in the patients with SAP. Furthermore, there are growing evidences for the monomers isolated from the herbs of CQCQD decoction that (1) saikosaponin A, baicalin, and chlorogenic acid could inhibit some proinflammatory signal pathway to ameliorate inflammatory cascade reaction [26–28]; (2) saikosaponin C and baicalein could inhibit LPS-induced apoptosis in human umbilical vein endothelial cells [29, 30]; (3) wogonin could inhibit LPS-induced vascular hyperpermeability via inhibiting cytoskeleton reorganization [31]. Consequently, we speculated that the treatment of CQCQD decoction in the patients with SAP endotoxemia-induced ALI could potentially ameliorate pulmonary albumin leak via depressing the inflammatory cascade reaction and alleviating the damage to cell-cell junction. However, the potential molecular mechanisms have not been systematically studied.

In this study, microarray and Western blot, and so forth, analyses were employed to reveal the key molecular mechanisms for CQCQD decoction in alleviating the pulmonary albumin leakage caused by endotoxemia in SAP rats.

## 2. Materials and Methods

### 2.1. Animals and Cell Culture

Adult Sprague-Dawley rats (250–300 g) were purchased from the Laboratory Animal Centre of Huaxi, Sichuan University (Chengdu, China). Animal experiments performed in this study were approved by the Institutional Animal Care and Use Committee of Sichuan University, and the corresponding license number was SCXK (Sichuan) 2014~09. Human umbilical vein endothelial cells (HUVECs) were obtained from Molecular Biology Lab of Sichuan University (Chengdu, China). HUVECs were cultured in DMEM with 10% fetal bovine serum (FBS), 100 U/mL penicillin, and 100 mg/mL streptomycin (Sigma-Aldrich, MO, USA) at 37°C with 5% CO_2_.

### 2.2. Preparation of CQCQD and Rat CQCQD-Containing Serum and Control Serum

**Figure 1:**
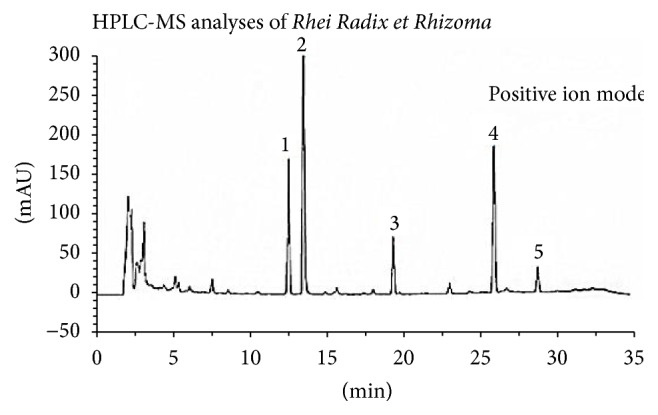
Chromatographic fingerprint of Rhei Radix et Rhizoma.

The Chinese drug decoction pieces in the CQCQD formula (Table 1) were acquired according to the prescribed proportions (% w/w) through the Pharmaceutical Preparation Section, West Chinese Hospital of Sichuan University, Chengdu, China. Each botanical name of these Chinese drug decoction pieces (Table 1) has been checked with http://www.theplantlist.org/ mentioning the data of accessing that website. According to the macroscopic and microscopic means mentioned in Chinese pharmacopoeia, the botanical authentication of each Chinese drug decoction piece has been performed by their manufacturers. In addition, the chemical characterization of major active ingredients of these Chinese drug decoction pieces (showed in Figure 1 for Rhei Radix et Rhizoma as an example) was identified by HPLC-MS analyses, adopting one or more reference marker compounds, to engender characteristic qualitative profiles (fingerprints). The assays of the quality control data of each Chinese drug decoction piece (heavy metal, pesticide residues, mycotoxins, impurity, ash, water content, extract, and the quantitative analysis of major active ingredients) were performed according to official pharmacopoeial methods and have been determined to be consistent with or better than pharmacopoeial standards as described previously.

Number in the bracket is the relative retention of the peak to the marker peak: (1) 0.65, Aloe-emodin, (2) 0.70, Rhein, (3) 1.00, Emodin, (4) 1.34, Chrysophanol, and (5) 1.49, Physcion.

Except Natrii Sulfas, the other seven dried components of Chinese drug decoction pieces were immersed in cold water for 60 minutes and then individually milled. Except Rhei Radix et Rhizoma, the other six milled components of the Chinese drug decoction pieces were admixed in the prescribed proportion (% w/w) as shown in Table 1 and then were extracted in 10-fold (w/v) of purified water by the decocting method for two times (40 minutes/each time) according to good manufacturing practices (GMP) at the College of the Pharmacy of Sichuan University. At the last ten minutes in the second decocting extraction, the milled component of Rhei Radix et Rhizoma drug decoction pieces was added to the extract liquids according to the prescribed proportion (% w/w) as shown in Table 1 for further decocting extraction. Afterwards, the mixing extracts of the seven Chinese drug decoction pieces and the pure product of Natrii Sulfas were admixed according to the prescribed proportion (% w/w) as shown in Table 1. The extracts were filtered, concentrated, spray-dried, and lyophilized to obtain a powder (28.44% yield w/w based on the raw materials of Chinese drug decoction pieces). The aliquots were manufactured into clinical product through the addition of water-soluble sodium carboxymethyl starch (excipient) according to the proportion of 1 : 1 (% w/w). Before administration to animals, the lyophilized powder of CQCQD was prepared to a concentration of 2.4 g/mL of crude herbs/aqua pro injectione. Rat medicated serums were prepared according to published protocols [32]. Briefly, 10 rats were randomly divided into blank group and CQCQD group. Rats in CQCQD group were treated with CQCQD (0.1 mL/10 g b.w.×1 time/2 hrs, 6 times) by intragastric administration, while rats in blank group only received the same amount of normal saline. After the last administration, blood from the rats was collected and centrifuged to prepare CQCQD-containing serum and control serum. Finally, the serums were stored at −20°C prior to use. The previous report indicated that CQCQD-containing serum at the concentration of 10% was more effective than that at 5% in improving the viability of pancreatic acinar cells in SAP rats [33]. It can be concluded that highly efficient concentration of CQCQD-containing serum would probably be considered as 10%. Therefore, the CQCQD-containing serum or control serum was diluted to the concentration of 10% by using DMEM medium, respectively.

### 2.3. Experimental Design

For in vivo trial, rats were randomly divided into sham-operation (SO), SAP, and CQCQD group (*n* = 20/group). In this paper, “SAP group” was used to represent “SAP endotoxemia-induced ALI model group.” SAP endotoxemia-induced ALI model was induced by retrograde main pancreatic duct injection of 5% sodium taurocholate (1 mL/kg b.w.) combined with cecal ligation-perforation (CLP). At 1 hr after operation, rats in CQCQD group were treated with CQCQD (0.1 mL/10 g b.w.×1 time/2 hrs, 6 times) by intragastric administration, while rats in the other two groups only received the same amount of saline. At 1 hr before the end of experiment, 5 rats from each group received intravenous injection of 2% Evans blue (EB) (2 mL/kg). At 12 hours after operation, blood samples were collected. Then using Infusion Pump (Fresenius Vial SA, France), the blood in the pulmonary circulation was cleared away. After that, the tissues on the edge of 0.5 cm in the lungs of rats in each group were collected. For in vitro trial, according to our research targets, HUVECs were divided into the following six groups: (1) Normal group (pretreated with normal rat serums for 1 hr and then added DMEM medium with 10% FBS and coincubated for 12 hrs); (2) LPS group (pretreated with normal rat serums for 1 hr and then added 1 *μ*M LPS and coincubated for 12 hrs); (3) CQCQD group (pretreated with CQCQD medicated serums for 1 hr and then added 1 *μ*M LPS and coincubated for 12 hrs); (4)–(6) selective inhibition agents groups (pretreated with normal rat serums + inhibitors of c-Src (PP1, 10 *μ*M) or PI3K (LY294002, 30 *μ*M) or transfected with siRNAs of c-Fos and then added 1 *μ*M LPS and coincubated for 12 hrs).

### 2.4. Gene Expression Profiling and Data Analysis

The SO group, SAP group, and CQCQD group were represented by the letters N, S, and C, respectively. The gene expression profiles of lung tissue in each sample were determined using Rat OneArray® Plus Microarray (Phalanx Biotech, Taiwan). The data of Rat OneArray Plus were analyzed using Rosetta Resolver® System (Rosetta Biosoftware), Annotation ROA2 release 1.0 (databases: NCBI RefSeq release 65 and Ensemble release 76 cDNA sequences Rnor_5.0 annotations), and R 3.0.3 software (http://www.r-project.org/). DEGs were identified as *P* < 0.05 and |log⁡2(Ratio)|≧0.585 by unpaired* t*-test. The functional enrichment analyses of KEGG_pathways associated with the lists of genes were generated by WebGestalt (http://www.webgestalt.org/) (*P* < 0.05 and entitles ≥3).

### 2.5. Protein-Protein Interaction (PPI) Network Construction and Hub Proteins Identification

The list of the DEGs, selected according to the study objective, was input into NetworkAnalyst software (Hancock Lab, Canada). The proteins, encoded by the prioritized and training DEGs, were employed as the initial seed proteins to interact with other essential proteins to construct the PPI network. The nodes with high degrees and betweenness would be considered as the hub nodes.

### 2.6. Measurement of Plasma LPS, sCd14 (Soluble-Monocyte Differentiation Antigen CD14), and Lbp (Lipopolysaccharide-Binding Protein)

Plasma LPS were measured by Limulus Amebocyte Lysate assay (Lonza, MD, USA) according to the manufacturer's protocol, and the sCd14 and Lbp levels in plasma were measured using ELISA kits (R&D, Minnesota, USA).

### 2.7. Assays of Amylase and Albumin in Sera as well as PaO_2_, PaCO_2_, and SpO_2_

The amylase and albumin in sera were measured by the fully automatic biochemical analyzer (TBA-120FR, Toshiba Medical Systems Corporation, Tochigi, Japan). PaO_2_, PaCO_2_, and SpO_2_ were detected by the blood-gas analyzer (RAPIDPOINT 500, Siemens Healthcare Diagnostics Inc., New York, USA).

### 2.8. Determination of the Total Protein Levels, Total Cell Counts, and the Concentration of TNF-*α*, IL-6, and IL-1*β* in the Bronchoalveolar Lavage Fluid (BALF)

The left lungs were lavaged via the bronchus with 1.5 mL PBS for three times; after that the BALF was collected for detecting total protein levels (Bicinchoninic Acid Kit for Protein Determination, Sigma-Aldrich, MO, USA) and total cell counts (Wright-Giemsa staining, Shanghai Haling Biotechnology Co. Ltd., Shanghai, China) as well as the concentrations of TNF-*α*, IL-6, and IL-1*β* (ELISA kits, R&D Systems, Minneapolis, MN, USA) according to the instructions of manufacturers.

### 2.9. Measurement of Albumin Transcapillary Escape Rate (TER)

EB would bind tightly to sera albumin and act as a hypersensitive marker for albumin leak [34]. After the operation immediately, from each group 5 rats were randomly selected and received caudal vein injection of EB (1 mg/mL) according to the concentration of 0.2 mg/kg. Before the injection of EB, blood samples of 1 mL were drawn and centrifuged for 10 min (3000 rpm) to remove the supernatant; after that, the absorbance of blank plasma at 620 nm and 740 nm was detected by a spectrophotometer (Beijing Analysis Instrument Co. Ltd., Beijing, China), to establish the standard curve of relationship between EB concentration and EB620corr (a correction value of plasmatic absorbance at 620 nm by the EB). Then, after injection of EB, as well as 6 hrs after operation and also at the end of the experiment, the plasmatic absorbance at 620 nm and 740 nm in EB-treated rats was assayed to calculate TER value according to the attenuation law of EB620coor of samples, which was based on the standard curve.

### 2.10. Measurement of Pulmonary EB Content

The EB contents in tissues are positively correlated with the quantity of the albumin leak [34]. The lung tissues from each group were soaked in N,N-dimethylformamide. Homogenate was incubated and centrifuged for detecting the absorbance at 620 nm, and the EB concentration was measured based on a standard curve of EB-N,N-dimethylformamide solutions.

### 2.11. Measurement of Pulmonary Water Content

 From each group 5 rats were randomly selected, and the right lung tissues of these rats were obtained, and then we measured, respectively, the wet and dry weight of the lungs, to calculate the pulmonary water content according to the research by Xu et al. [35].

### 2.12. HE Staining

The pancreatic and pulmonary samples were fixed in 4% paraformaldehyde and subjected to the staining of haematoxylin and eosin. For each HE stained slice, 10 visual fields under a high-power microscope (×400) were randomly selected and scored by one specific pathologist.

### 2.13. Transient Transfection with siRNAs

The small interfering RNA (Stealth siRNA) for Human c-Fos (HSS103799, HSS103800, and HSS177475) and scrambled siRNA were from Thermo Fisher Scientific Inc. (MA, USA). Transient transfection of siRNAs was performed according to the manufacturer's instructions.

### 2.14. Transwell® Assays

HUVECs were seeded on 24 mm Transwell with 0.4 *μ*m Pore Polyester Membrane Insert, Sterile (Corning, MA, USA), and grown until confluency. HUVECs monolayers of TEER were measured by Millicell® electrical resistance apparatus (Millipore, FL, USA) and TEER values were calculated in accordance with the formula as described previously [36]. To further measure paracellular permeability, FITC-dextran (50 g/mL) was added to the upper chambers of the Transwell and coincubated with cell monolayer at 37°C for 60 min, and then samples were extracted from both the upper and lower chambers for fluorescence determination at 535 nm; furthermore, the permeability to dextran was calculated as described previously [37].

### 2.15. Determination of the Concentrations of TNF-*α*, IL-6, and IL-1*β* in the Supernatant of HUVECs

After centrifugation, the supernatant of HUVECs was collected for detecting the concentrations of TNF-*α*, IL-6, and IL-1*β* (ELISA kits, R&D Systems, Minneapolis, MN, USA) according to the instructions of manufacturers.

### 2.16. Western Blot Analysis

Total protein in the three groups of HUVECs was separated by 10% acrylamide SDS-PAGE and transferred onto Immobilon® PVDF membranes (Sigma-Aldrich, MO, USA). Then the membranes were blocked and incubated with specific primary antibodies in 4°C overnight (1 : 1000 of dilution) followed by incubation with a secondary antibody. Membranes-bound antibodies were tested by Amersham*™* ECL*™* (Sigma-Aldrich, MO, USA).

### 2.17. Statistical Analysis

Data are shown as mean ± standard deviation (SD). Dunnett's pairwise multiple comparison* t*-test was used to examine the significant differences by SPSS 19.0 software. Statistical difference was accepted at *P* < 0.05.

## 3. Results

### 3.1. Identification of DEGs in the S/N and C/S DEGs Clusters

According to the Rosetta Biosoftware, out of 20715 gene probes on the Rat OneArray Plus Microarray, 3190 gens (1678 genes upregulated and 1512 genes downregulated) in S/N DEGs cluster and 4138 genes (2404 genes upregulated and 1734 genes downregulated) in C/S DEGs cluster were differentially expressed (*P* < 0.05, |log⁡2(Ratio)|≧0.585).

### 3.2. Predictions of the Target Proteins for CQCQD to Attenuate Pulmonary Albumin Leakage Based on the Analyses of Rats Pulmonary Tissue Microarray and Also the Construction of PPI Network

**Figure 2:**
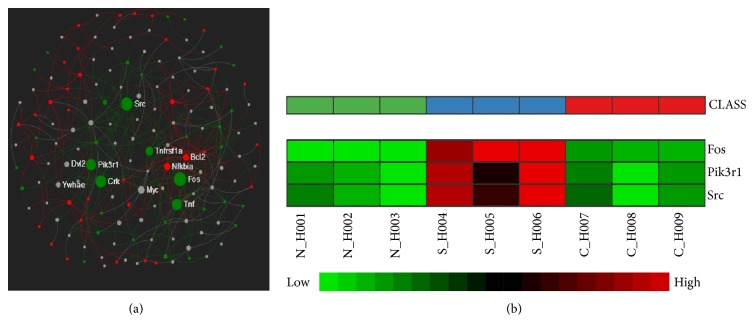
Predicting target proteins for CQCQD to attenuate pulmonary albumin leakage based on the analyses of rats pulmonary tissue microarray and also the construction of PPI network. (a) The proteins, encoded by these prioritized hub nodes including Fos, Pik3r1, and Src, were predicted as the key target proteins (red nodes = upregulated DEGs, green nodes = downregulated DEGs, grey nodes = other interactive genes; the size of nodes = the betweenness of the node). (b) The expressions of these 3 prioritized hub nodes in each sample within each group (red represents upregulation and green represents downregulation).

The KEGG_pathway enrichment analyses closely related to paracellular leakage-dependent albumin leakage provided the important evidences that some of the DEGs in C/S and S/N DEGs clusters were significantly enriched in these 5 pathways including inflammatory response (Toll-like receptor signaling pathway), paracellular leakage (adherens junction, tight junction, and regulation of actin cytoskeleton), and apoptosis (apoptosis) (*P* < 0.05, entitles ≧3, Table 2). And then, the DEGs (C/S DEGs cluster), enriched in these KEGG_pathways, were inputted into NetworkAnalyst database to construct a PPI network (nodes = 623, edges = 868, and seed proteins = 72). The top three nodes with the highest degree and betweenness (degree >40 and betweenness >1000) were Fos (protooncogene c-Fos), Pik3r1 (phosphatidylinositol 3-kinase regulatory subunit alpha), and Src (protooncogene tyrosine-protein kinase Src), respectively (Figure 2(a)). Therefore, the 3 proteins including c-Fos, c-Src, and p85*α*, encoded by these 3 genes, were considered as the prioritized hub proteins and also predicted as the key target proteins for CQCQD to attenuate pulmonary albumin leakage. Furthermore, these 3 genes displayed the expression patterns of C/S reversing S/N (*P* < 0.05, |log⁡2(Ratio)|≧0.585), which were shown in the heatmap (Figure 2(b)).

### 3.3. Effect of CQCQD on the Characteristics Indexes Closely Related to SAP Complicated by Endotoxemia

As shown in Table 3, the blood concentrations of LPS, sCd14, Lbp, and amylase in SAP group were remarkably higher than that in SO group (^▲▲^ *P* < 0.01); strikingly, the blood concentrations of LPS, sCd14, Lbp, and amylase in CQCQD group were obviously decreased compared with that in SAP group (^△^ *P* < 0.05 or ^△△^ *P* < 0.01).

### 3.4. Effect of CQCQD on the Characteristics Indexes Closely Related to Systemic and Pulmonary Albumin Leakage

As shown in Table 4, the TER as well as pulmonary EB and water contents in SAP group were remarkably higher than those in SO group; meanwhile, the serum albumin in SAP group was significantly lower than that in SO group (^▲▲^ *P* < 0.01); fortunately, the treatment of CQCQD had significantly reversed the changes in these characteristics indexes compared with that in SAP group (^△^ *P* < 0.05 or ^△△^ *P* < 0.01).

### 3.5. Effect of CQCQD on the Airways Indicators and Respiratory Function

As shown in Table 5, acting as the airways indicators, the total protein levels and total cell counts as well as the levels of TNF-*α*, IL-6, and IL-1*β* of BALF in SAP group were remarkably higher than that in SO group (^▲▲^ *P* < 0.01); acting as the characteristics indexes of respiratory function, PaCO_2_ in SAP group was markedly higher than that in SO group; meanwhile, PaO_2_ and SaO_2_ in SAP group were evidently lower than those in SO group (^▲▲^ *P* < 0.01); fortunately, the treatment of CQCQD had significantly reversed the changes in these characteristics indexes compared with that in SAP group (^△^ *P* < 0.05 or ^△△^ *P* < 0.01).

### 3.6. Effect of CQCQD on the Pancreatic and Pulmonary Histopathological Changes

**Figure 3:**
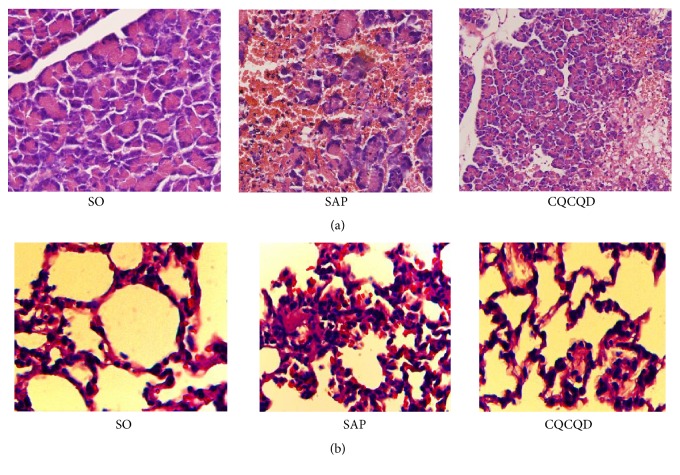
HE staining. (a) Pancreatic histopathological changes (×200) (pancreatic histopathological score: SO = 0.32 ± 0.08, SAP = 16.85 ± 2.89, CQCQD = 8.87 ± 1.26; SAP versus SO, *P* < 0.01, CQCQD versus SAP, *P* < 0.01); (b) pulmonary histopathological changes (×400) (pulmonary histopathological score: SO = 0.45 ± 0.12, SAP = 3.44 ± 0.46, CQCQD = 1.87 ± 0.35; SAP versus SO, *P* < 0.01, CQCQD versus SAP, *P* < 0.05).

**Figure 4:**
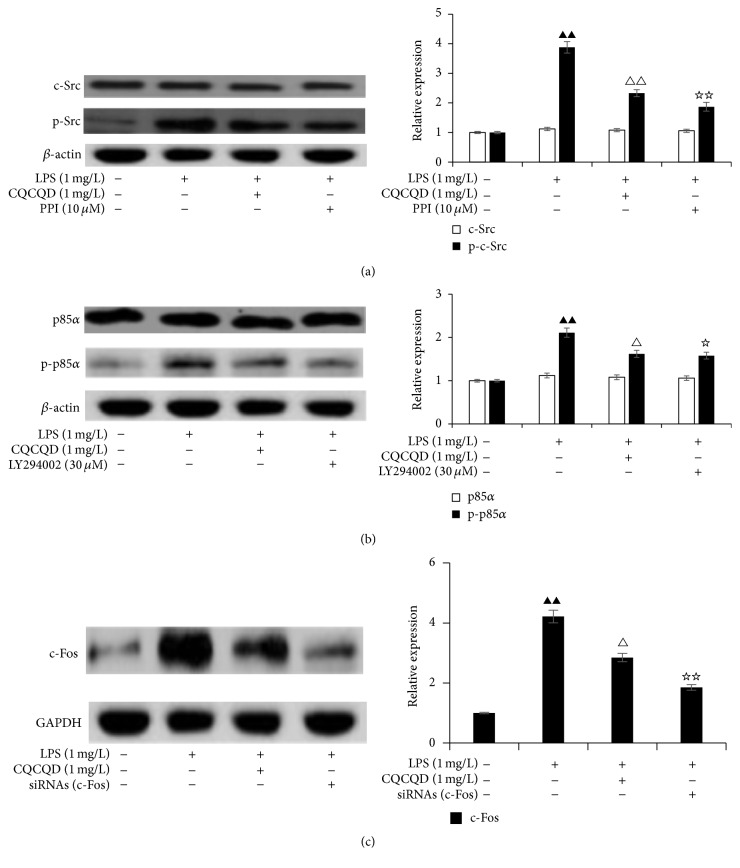
The expression changes in these 3 predicted proteins in HUVECs. ^▲▲^ *P* < 0.01 versus Normal group, ^△△^ *P* < 0.01 versus LPS group, ^△^ *P* < 0.05 versus LPS group, ^☆☆^ *P* < 0.01 versus LPS group, and ^☆^ *P* < 0.05 versus LPS group.

The pancreatic and pulmonary histopathological changes were shown as follows: (1) the edema, hemorrhage, massive inflammatory cells infiltration, and a large number of pancreatic acinar cells' necrosis were seen in the pancreatic tissue of SAP rats (Figure 3(a)); (2) the broken alveolar structure, thickened alveolar wall, and the infiltration of massive inflammatory cell and red blood cells were shown in SAP rats (Figure 3(b)); (3) nevertheless, the therapy of CQCQD decoction effectively alleviated the pancreatic and pulmonary histopathological changes compared with that in SAP group (Figure 3); (4) according to the above histopathological changes, the pancreatic and pulmonary histopathological scores of rats in SAP group were evidently higher than that in SO group (*P* < 0.01), while those in CQCQD group were visibly decreased compared with that in SAP group (*P* < 0.05) (Figure 3).

### 3.7. Effect of the Pretreatment of CQCQD on LPS-Mediated Changes in TEER, Paracellular Leakage, and Proinflammatory Cytokines Release in HUVECs Imitating the Actions of the Selective Inhibitions Agents of These Predicted Proteins

As shown in Table 6, LPS-stimulated HUVECs show the remarkable increase in proinflammatory cytokines release and dextran flux (paracellular leakage) compared to that in Normal group (*P* < 0.05); meanwhile, TEER in LPS group was significantly lower than that in Normal group (*P* < 0.05). According to the analyses results of the PPI network constructed by the analyses of rats' pulmonary tissue microarray, c-Fos, c-Src, and p85*α* were predicted as the key target proteins for CQCQD in alleviating pulmonary albumin leakage. Additional information: p85*α* is encoded by Pik3r1 [38], c-Fos is encoded by Fos [39], and c-Src is encoded by Src [40]. In order to verify these predictions that firstly required demonstrating the important involvements of these 3 predicted target proteins in LPS-mediated changes in TEER, paracellular leakage, and proinflammatory cytokines release in HUVECs, HUVECs were pretreated with the inhibitors of c-Src (PP1) or PI3K (LY294002) or transfected with siRNAs of c-Fos, which provided the important evidences that the pretreatment with PP1 or LY294002 as well as the transfection with siRNAs of c-Fos remarkably reversed LPS-induced increases in proinflammatory cytokines release and paracellular leakage and also the decrease in TEER (*P* < 0.05, Table 6). Strikingly, the pretreatment of CQCQD imitated the actions of the selective inhibitions agents of these predicted proteins to significantly reverse LPS-mediated increase in paracellular leakage and proinflammatory cytokines release as well as the decrease in TEER (*P* < 0.05, Table 6).

### 3.8. Effects of the Pretreatment of CQCQD on the LPS-Induced Expression Changes in These 3 Predicted Proteins in HUVECs Imitating the Actions of the Selective Inhibitions Agents of These Predicted Target Proteins

**Figure 5:**
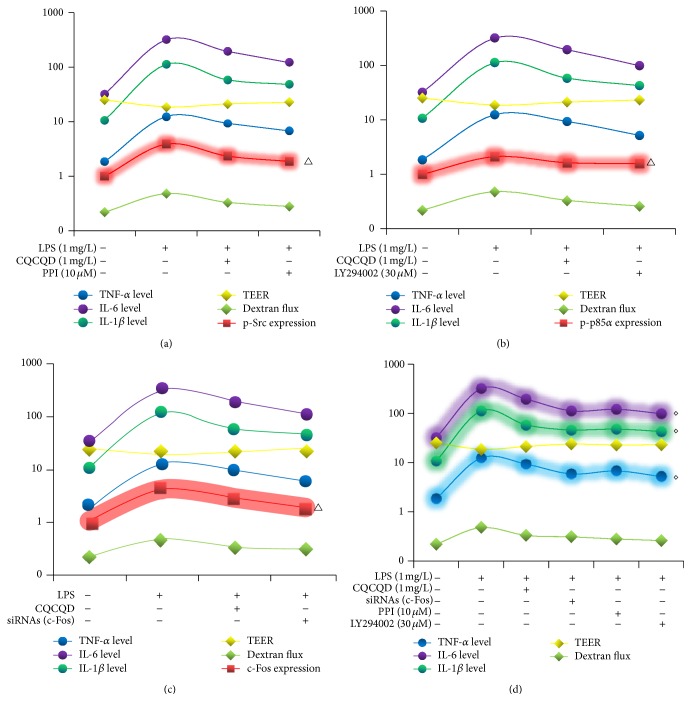
Correlations among the expressions of p-Src, p-p85*α*, and c-Fos and LPS-induced paracellular leakage and the change in TEER as well as LPS-induced proinflammatory cytokines release in HUVECs (benchmark logarithmic scale = 10; ^△^ *ρ* > 0.98 versus dextran flux, or the levels of TNF-*α* or IL-6 or IL-1*β*, ^△^ *ρ* < −0.98 versus TEER, ^*♢*^ *ρ* > 0.98 versus dextran flux, and ^*♢*^ *ρ* < −0.98 versus TEER).

Since the selective inhibitions agents of these predicted target proteins show the significant alleviating effects on LPS-mediated paracellular leakage and proinflammatory cytokines release in HUVECs, the analyses of the expression of these predicted target proteins were required to reveal the molecular mechanisms. HUVECs were treated as described previously, and then Western blot analyses were performed. This study provided the important evidences that (1) LPS observably induced the high expressions of p-Src, p-p85*α*, and c-Fos compared with that in Normal group (*P* < 0.05, Figure 4) but did not induce the significant changes in expression of nonphosphorylated Src and p85*α* compared with that in Normal group (*P* > 0.05, Figures 4(a) and 4(b)); (2) the pretreatment with the inhibitors of c-Src (PP1) remarkably inhibited LPS-induced high expression of p-Src (*P* < 0.05, Figure 4(a)) but did not induce the significant change in expression of nonphosphorylated Src compared with that in Normal or LPS group (*P* > 0.05, Figure 4(a)); (3) the pretreatment with the inhibitors of PI3K (LY294002) markedly suppressed LPS-induced high expression of p-p85*α* (*P* < 0.05, Figure 4(b)) but did not trigger the significant change in expression of nonphosphorylated p85*α* compared with that in Normal or LPS group (*P* > 0.05, Figure 4(b)); (4) the transfection with siRNAs of c-Fos evidently inhibited LPS-induced overexpression of c-Fos (*P* < 0.05, Figure 4(c)); (5) strikingly, the pretreatment of CQCQD on HUVECs successfully imitated the actions of the selective inhibitions agents in the same way to inhibit LPS-mediated high expressions of p-Src, p-p85*α*, and c-Fos (*P* < 0.05, Figure 4) and likewise did not induce the significant changes in expressions of nonphosphorylated Src and p85*α* compared with that in Normal or LPS group (*P* > 0.05, Figures 4(a) and 4(b)). Therefore, the inhibition effects of LPS-mediated high expressions of p-Src, p-p85*α*, and c-Fos were the key molecular mechanisms for CQCQD or the selective inhibitions agents acting on these 3 predicted target proteins.

### 3.9. Correlations among the Expressions of p-Src, p-p85*α*, and c-Fos and LPS-Induced Paracellular Leakage and the Change in TEER as well as LPS-Induced Proinflammatory Cytokines Release in HUVECs

According to the benchmark logarithmic scale of 10, the line charts had been formed, and then the correlational analyses were performed among the expressions of p-Src, p-p85*α*, and c-Fos and LPS-induced paracellular leakage and the change in TEER as well as LPS-induced proinflammatory cytokines release in HUVECs. The*ρ* value of correlation coefficient is calculated based on the original data of these parameters, but not the logarithmic data. This study provided the first evidences that (1) the expressions of p-Src, p-p85*α*, and c-Fos were positively correlated with LPS-induced paracellular leakage and proinflammatory cytokines release (*ρ* > 0.98) and negatively correlated with LPS-induced changes in TEER (*ρ* < −0.98), which demonstrated the very highly linear regression correlation (Figures 5(a), 5(b), and 5(c)); (2) there were also the remarkably positive correlations between LPS-induced proinflammatory cytokines release and LPS-induced paracellular leakage (*ρ* > 0.98) and also the notably negative correlations between LPS-induced proinflammatory cytokines release and LPS-induced changes in TEER (*ρ* < −0.98), which show the highly linear regression correlation (Figure 5(d)).

## 4. Discussion

The methods of intraperitoneal (IP) or intratracheal (IT) injection of LPS-inducing lung injury are simple, but they cannot simulate the natural course of SAP endotoxemia-induced ALI [41]. Therefore, we used the methods of retrograde main pancreatic duct injection of 5% sodium taurocholate combined with cecal ligation-perforation to establish SAP endotoxemia-induced ALI model. After cecal ligation, the feces were accumulated in cecum, and then, the puncture by using 18# needle was performed on the ligated cecum to form 3 holes. After that, the mixed bacteria in the intestinal contents constantly enter into the abdominal cavity to induce the infection of the abdominal cavity, which would result in endotoxemia or sepsis, and eventually initiate pulmonary albumin leakage to trigger ALI. Hence, the models, induced by retrograde main pancreatic duct injection of sodium taurocholate combined with cecal ligation-perforation, could realistically demonstrate the pathophysiological process of SAP endotoxemia-induced ALI that is more close to the true process in clinic [41]. Strikingly, retrograde main pancreatic duct injection of 5% sodium taurocholate combined with cecal ligation-perforation successfully induced SAP complicated with endotoxemia (Table 3), the enhancement to systemic and pulmonary albumin leak (Table 4), depravation of airways indicators (Table 5), respiratory dysfunction (Table 5), and also the adverse change in pancreatic and pulmonary tissue pathology (Figure 3), which suggested that the models of ALI caused by endotoxemia in SAP rats were successfully constructed. Fortunately, the therapy of CQCQD showed the protective effects on the rats of SAP endotoxemia-induced ALI via significantly ameliorating the changes in the above characteristics indexes (Tables 3, 4, and 5 and Figure 3).

The pathophysiological mechanisms of alleviating paracellular leakage-dependent albumin leak mainly include (1) inhibiting the excessive reassembly of F-actin and the reorganization of cytoskeleton, (2) suppressing the disassembly of TJs, AJs, and F-actin at cell-cell contact sites, (3) reassembling TJs, AJs, and F-actin at damaged cell-cell contact sites, (4) maintaining AJs, TJs, and the homeostasis of actin, (5) suppressing apoptosis, and (6) inhibiting inflammatory response [42]. In our study, the analyses of rats pulmonary tissues microarray and also the construction of PPI network provided the important evidences that (1) some DEGs in C/S and S/N DEGs clusters were significantly enriched in the 5 KEGG pathways closely related to the above pathophysiological mechanisms (Table 2); (2) there were the 3 prioritized hub proteins (c-Fos, c-Src, and p85*α*) to be predicted as the key target proteins for CQCQD in alleviating pulmonary albumin leakage, and these 3 genes displayed the expression patterns of C/S reversing S/N (Figure 2). In order to verify these predictions that firstly required demonstrating the important involvements of these 3 predicted target proteins in LPS-mediated changes in TEER, paracellular leakage, and proinflammatory cytokines release in HUVECs and then further investigate the molecular mechanisms involved, our studies in vitro provided the several important evidences that (1) the selective inhibition agents of these 3 predicted target proteins remarkably reversed LPS-induced increases in proinflammatory cytokines release and paracellular leakage as well as the decrease in TEER (Table 6); (2) the inhibition effects of LPS-mediated high expressions of p-Src, p-p85*α*, and c-Fos were the key molecular mechanisms for the selective inhibitions agents acting on these predicted target proteins (Figure 4); (3) the expressions of p-Src, p-p85*α*, and c-Fos were highly and positively correlated with LPS-induced paracellular leakage and proinflammatory cytokines release and highly and negatively correlated with LPS-induced changes in TEER (Figures 5(a), 5(b), and 5(c)), which suggested that inhibitions of LPS-mediated high expressions of p-Src, p-p85*α*, and c-Fos could suppress LPS-induced paracellular leakage and proinflammatory cytokines release. And then, our in vitro studies successfully confirmed these predictions: (1) the pretreatment of CQCQD on LPS-stimulated HUVECs which imitated the actions of the selective inhibitions agents to inhibit LPS-induced high expressions of p-Src, p-p85*α*, and c-Fos (Figure 4); (2) ultimately, significantly alleviated LPS-mediated increase in paracellular leakage and proinflammatory cytokines release as well as the decrease in TEER (Table 6). The above evidences suggested that CQCQD could probably attenuate the paracellular leakage-dependent albumin leak via reversing endotoxemia-triggered expressions changes in these predicted target proteins. The potential molecular mechanisms were further analyzed as follows. 

*Alleviating Paracellular Leakage via Inhibiting the Expressions of Upstream Trigger Molecules of Toll-Like Receptors Pathway and Inflammatory Response.* LPS would activate distinct pathway to induce inflammatory response via Toll-like receptors cascades, and Lbp and Cd14 are key upstream molecules that are common to all TLRs [43]. Fortunately, our study in vivo demonstrated that the therapy of CQCQD significantly decreased the levels of sera amylase and plasma LPS, Lbp, and Cd14 compared to that in SAP group (Table 3), which suggested that the therapy of CQCQD could attenuate the increase in the upstream trigger molecules caused by SAP complicated by endotoxemia. The activation of Toll-like receptors cascades would induce the upregulation of p-Src to phosphorylate p85*α* and induce the activation of Akt (kinase Serine/threonine-protein), which ultimately leads to the activation of NFKB and MAPK cascades and initiates the excessive transcriptions of proinflammatory cytokines [44, 45]. c-Fos is a key transcription factor in MAPK cascade, whose overexpression would also mediate the numerous transcriptions of inflammatory cytokines [46]. These previous reports have been confirmed by our in vitro studies results that inhibitions of LPS-mediated high expressions of p-Src, p-p85*α*, and c-Fos could suppress LPS-induced proinflammatory cytokines release (Figures 4, 5(a), 5(b), and 5(c) and Table 6), and the specific analyses have been demonstrated in the first paragraph of the Discussion. Strikingly, the pretreatment of CQCQD imitated the actions of these selective inhibitions agents to inhibit LPS-mediated high expressions of p-Src, p-p85*α*, and c-Fos, therefore suppressing the proinflammatory cytokines release (Figure 4 and Table 6). It is well known that the increase in proinflammatory cytokines release will lead to the enhancement of the albumin leakage induced by LPS. Our study in vitro provided the important evidences that there were also remarkably positive correlations between LPS-induced proinflammatory cytokines release and paracellular leakage (Figure 5(d)), which suggested that the pretreatment of CQCQD could probably contribute to alleviating LPS-induced paracellular leakage via inhibiting proinflammatory cytokines release (Table 4).

*Protections and Maintenance of AJs, TJs, and the Homeostasis of F-Actin.* LPS-stimulated HUVECs had significantly induced the upregulated expressions of p-Src, p-p85*α*, and c-Fos (Figure 4), which would probably induce the disassembly of AJs, TJs, and F-actin at cell-cell contact sites and trigger the excessive reassembly of F-actin and cytoskeleton reorganization. The molecular mechanisms were described as follows: (1) the high expressions of p-Src at Tyr-419 would increase kinase activity to phosphorylate downstream molecules [47], which would probably induce (a) the high phosphorylation of p85*α* to aggrandize the Akt (Serine/threonine-protein kinase) activation to mediate the reorganization of cytoskeleton [48], (b) the Tjp1 Tyr hyperphosphorylation to mediate the collapse of TJs [49], and (c) the high phosphorylation of Ctnnb1 (Catenin beta-1) and Ctnnd1 (Catenin delta-1) to induce disassembly of AJs [50, 51]; (2) the high expressions of p-p85*α* would enhance Akt activity to induce cytoskeleton reorganization [48]; (3) the excessive reassembly of F-actin would further induce the overexpression and activation of Fos [52], which would mediate more transcriptions of inflammatory cytokines, thus forming a vicious cycle. These inferences have been confirmed by the studies in vitro that LPS-stimulated HUVECs have markedly caused the decrease in TEER as well as the increase in proinflammatory cytokines release and paracellular leak (Table 6). Notably, the studies in vitro have provided the key evidences that inhibitions of LPS-mediated high expressions of p-Src, p-p85*α*, and c-Fos could reverse LPS-induced decrease in TEER and the increase in paracellular leak (Figures 4, 5(a), 5(b), and 5(c) and Table 6), and the specific analyses have been demonstrated in the first paragraph of the Discussion. More notably, the pretreatment of CQCQD imitated the actions of these selective inhibitions agents of these 3 predicted target proteins to inhibit LPS-induced high expressions of p-Src, p-p85*α*, and c-Fos (Figure 4). Consistent with this, the pretreatment of CQCQD evidently reversed LPS-induced decrease in TEER as well as the increase in proinflammatory cytokines release and paracellular leak (Table 6), which suggested that the pretreatment of CQCQD on LPS-stimulated HUVECs could probably protect and maintain AJs, TJs, and the homeostasis of F-actin.

To further verify the therapeutical effect of CQCQD on rats with SAP endotoxemia-induced ALI, our study provided the key evidences that, in in vivo study, the therapy of CQCQD significantly ameliorated the enhancements of systemic and pulmonary albumin leakage as well as the depravation of airways indicators (Tables 4 and 5) and ultimately improved respiratory dysfunction and pancreatic and pulmonary histopathological changes (Table 4 and Figure 3).

In conclusion, the therapy of CQCQD could alleviate the LPS- and cytokine-mediated pulmonary albumin leakage, which were mainly via inhibiting the upregulation of p-Src, p-p85*α*, and c-Fos.

## Figures and Tables

**Table 1:** Components of Chinese drug decoction pieces in the CQCQD formula and their production lot numbers and the relative contents of major active ingredients.

Pharmaceutical name and Chinese phonetic name	Botanical name and plant part	Composition (%)	Lot number	Quantitative analysis of major active ingredients	
				Reference markers (HPLC)	Relative content (% w/w)
Bupleuri Radix (Chai Hu)	*Bupleurum chinense *DC (Apiaceae), root	11.111	2014100802	Saikosaponin a	0.383–0.421
				Saikosaponin d	0.381–0.398

Scutellariae Radix (Huang Qin)	*Scutellaria baicalensis *Georgi (Lamiaceae), root	11.111	2014081003	Baicalin	12.338–14.321

Rhei Radix et Rhizoma (Da Huang)	*Rheum palmatum *L. (Polygonaceae)	14.814	2014080301	Aloe-emodin	0.348–0.367
				Rhein	1.002–1.162
				Emodin	0.513–0.628
				Chrysophanol	0.661–0.702
				Physcion	0.268–0.301

Natrii Sulfas (Mang Xiao)	*Mirabilite* (sulfate minerals), crystal	14.814	2014030506	Thenardite	99.812–99.993

Magnoliae Officinalis Cortex (Hou Pu)	*Magnolia officinalis *Rehder & E. H. Wilson (Magnoliaceae), root, branch, and stem bark	11.111	2014060501	Magnolol	2.118–2.491
				Honokiol	2.181–2.228

Aurantii Fructus Immaturus (Zhi Shi)	*Citrus aurantium *L. (Rutaceae), immature fruit	11.111	2014053101	Synephrine	0.612–0.641
				Hesperidin	1.231–1.445

Artemisiae Scopariae Herba (Yin Chen)	*Artemisia scoparia *Waldst. & Kitam. (Asteraceae), aerial part	11.111	2014030601	Chlorogenic acid	0.598–0.693
				Escoparone	0.598–0.6341

Gardeniae Fructus (Zhi Zi)	*Gardenia jasminoides* J. Ellis (Rubiaceae), ripe fruit	14.814	2014110301	Geniposide	5.989–6.959

**Table 2:** KEGG pathway enrichments analyses (closely related to albumin leakage) in the S/N and C/S DEGs cluster.

Pathway	Total	Hits	*P* value	FDR
Toll-like receptor signaling pathway	96	39	8.96*E* − 22	3.18*E* − 20
Tight junction	122	48	1.47*E* − 19	3.13*E* − 18
Adherens junction	71	27	1.97*E* − 15	1.75*E* − 14
Regulation of actin cytoskeleton	183	62	1.74*E* − 08	6.86*E* − 08
Apoptosis	82	25	6.07*E* − 08	2.23*E* − 07

**Table 3:** The blood concentrations of LPS, sCd14, Lbp, and amylase.

Indexes	SO group	SAP group	CQCQD group
LPS (ng/mL)	0.13 ± 0.04	0.52 ± 0.06^▲▲^	0.36 ± 0.09^△^
LBP (ug/mL)	2.46 ± 0.35	9.12 ± 0.61^▲▲^	7.48 ± 0.49^△^
sCD14 (ug/mL)	2.23 ± 1.24	7.08 ± 5.37^▲▲^	4.31 ± 4.02^△△^
Serum amylase (IU/L)	1480.76 ± 422.46	2583.96 ± 297.51^▲▲^	2039.23 ± 141.99^△△^

^▲▲^ *P* < 0.01 versus SO, ^△^ *P* < 0.05 versus SAP, and ^△△^ *P* < 0.01 versus SAP.

**Table 4:** The characteristics indexes closely related to systemic and pulmonary albumin leakage.

Indexes	SO	SAP	CQCQD
TER (%/h)	8.56 ± 1.14	24.73 ± 2.19^▲▲^	16.42 ± 1.45^△^
Serum albumin (g/L)	23.03 ± 0.31	13.26 ± 0.63^▲▲^	18.49 ± 0.38^△△^
Pulmonary EB content (ug/g)	37.29 ± 3.09	77.14 ± 9.24^▲▲^	61.03 ± 4.38^△^
Pulmonary water content (%)	71.23 ± 1.07	80.35 ± 1.37^▲▲^	75.01 ± 1.33^△^

^▲▲^ *P* < 0.01 versus SO, ^△^ *P* < 0.05 versus SAP, and ^△△^ *P* < 0.01 versus SAP.

**Table 5:** The airways indicators and respiratory function.

Indexes	SO	SAP	CQCQD
Total protein levels in BALF (mg/mL)	1.58 ± 0.16	9.34 ± 1.02^▲▲^	6.84 ± 0.72^△^
Total cell counts in BALF (×10^6^/L)	2.89 ± 0.06	9.82 ± 0.85^▲▲^	5.33 ± 0.21^△^
TNF-*α* levels in BALF (ng/L)	124.07 ± 17.35	189.61 ± 10.22^▲▲^	164.21 ± 7.88^△^
IL-6 levels in BALF (ng/L)	149.23 ± 15.47	217.27 ± 9.82^▲▲^	188.75 ± 11.49^△^
IL-1*β* levels in BALF (ng/L)	1068.93 ± 117.76	1992.80 ± 120.20^▲▲^	1567.14 ± 139.24^△^
PaCO_2_ (mmHg)	30.02 ± 0.98	47.16 ± 1.32^▲▲^	41.07 ± 1.08^△△^
PaO_2_ (mmHg)	105.87 ± 1.38	74.26 ± 1.33^▲▲^	90.25 ± 0.85^△△^
SaO_2_ (%)	98.66 ± 0.93	89.07 ± 0.74^▲▲^	97.82 ± 0.69^△△^

^▲▲^ *P* < 0.01 versus SO, ^△^ *P* < 0.05 versus SAP, and ^△△^ *P* < 0.01 versus SAP.

**Table 6:** The proinflammatory cytokines release, TEER, and paracellular leakage in HUVECs.

Indexes	Normal	LPS (1 mg/L)	CQCQD (1 mg/L)	Inhibition agents		
				siRNA (c-F-os)	PP1 (10 *μ*M)	LY294002 (30 *μ*M)
TNF-*α* (ng/mL)	1.85 ± 0.35	12.46 ± 0.41^▲^	9.32 ± 0.58^△^	5.85 ± 0.28^☆^	6.78 ± 0.31^⋄^	5.18 ± 0.33^¤^
IL-6 (*ρ*g/mL)	32.19 ± 7.55	318.45 ± 9.47^▲^	194.33 ± 8.61^△^	112.18 ± 9.98^☆^	121.23 ± 10.45^⋄^	98.56 ± 10.10^¤^
IL-1*β* (*ρ*g/mL)	10.75 ± 1.03	112.42 ± 4.55^▲^	58.16 ± 3.93^△^	45.78 ± 3.03^☆^	48.24 ± 3.56^⋄^	42.66 ± 3.75^¤^
TEER (Ω·cm^2^)	25.12 ± 1.66	18.61 ± 0.98^▲^	21.23 ± 1.12^△^	23.89 ± 1.03^☆^	22.64 ± 1.23^⋄^	23.13 ± 1.01^¤^
Dextran flux (cm/s)	0.22 ± 0.12	0.48 ± 0.29^▲^	0.33 ± 0.22^△^	0.31 ± 0.18^☆^	0.28 ± 0.16^⋄^	0.26 ± 0.14^¤^

^▲^ *P* < 0.05 versus Normal, ^△^ *P* < 0.05 versus LPS, ^☆^ *P* < 0.05 versus LPS, ^⋄^ *P* < 0.05 versus LPS, and ^¤^ *P* < 0.05 versus LPS.
